# Ulnar reconstruction in a multiply revised total elbow arthroplasty with temporary radial implantation of the ulnar component: a case report

**DOI:** 10.1016/j.xrrt.2026.100803

**Published:** 2026-06-18

**Authors:** Fabian Lanzerath, Tim Leschinger, Graham J.W. King

**Affiliations:** a Faculty of Medicine, Department of Orthopaedics and Trauma Surgery, University Hospital Cologne (Uniklinik Köln), University of Cologne, Cologne, Germany; b Roth | McFarlane Hand & Upper Limb Centre, St. Joseph's Health Care London, London, Ontario, Canada; c Department of Surgery, Western University, London, Ontario, Canada

## Introduction

Revision total elbow arthroplasty (TEA) for aseptic loosening in the setting of severe segmental bone loss is associated with some of the highest complication and reoperation rates in upper-extremity arthroplasty. In a systematic review, Geurts et al^5^ reported complication and reoperation rates of 44% and 22%, respectively.

Allograft–prosthesis composites (APCs) are well established in oncologic limb reconstruction and increasingly utilized for nononcologic elbows with massive bone loss.^1^^,^^4^^,^^12^ Midterm data, although very limited, demonstrate acceptable outcomes when precise graft preparation, cortical contact, and strut augmentation are achieved, though complications such as graft resorption, nonunion, and periprosthetic fracture remain common.^4^^,^^10^^,^^14^ Kamineni and Morrey reported that many proximal ulnar bone defects in revision TEA can be treated with strut allograft reconstruction, although restoration of deficient olecranon bone stock remained unreliable. More recently, Burnier et al^3^ reported revision elbow arthroplasty using a structural proximal ulnar allograft with attached triceps tendon for combined ulnar bone loss and triceps insufficiency in 10 elbows at a mean follow-up of 45 months; pain improved in all elbows, the mean flexion-extension arc was 95°, and the mean Mayo Elbow Performance Score (MEPS) was 76 points, although 6 elbows required reoperation.^9^

Modular distal humeral reconstruction systems provide a solution when metaphyseal bone is absent or nonviable, enabling intercalary replacement and reliable restoration of length and alignment.^7^ There are limited modular metallic options for bone deficiency in the proximal ulna.^2^

This case report describes a multiply revised total elbow reconstructed using a two-stage salvage strategy: (1) humeral APC reconstruction with temporary radial implantation of the ulnar component to restore a linked articulation while biologically rebuilding the ulna and (2) definitive reconstruction using a long-stem ulnar APC and a modular distal humeral replacement. No fixed time interval was defined for the second-stage reconstruction. The patient was followed clinically and radiographically, with a second stage reconstruction planned once mechanical failure of the radial component developed.

## Case presentation

### Patient history

**Figure 1:**
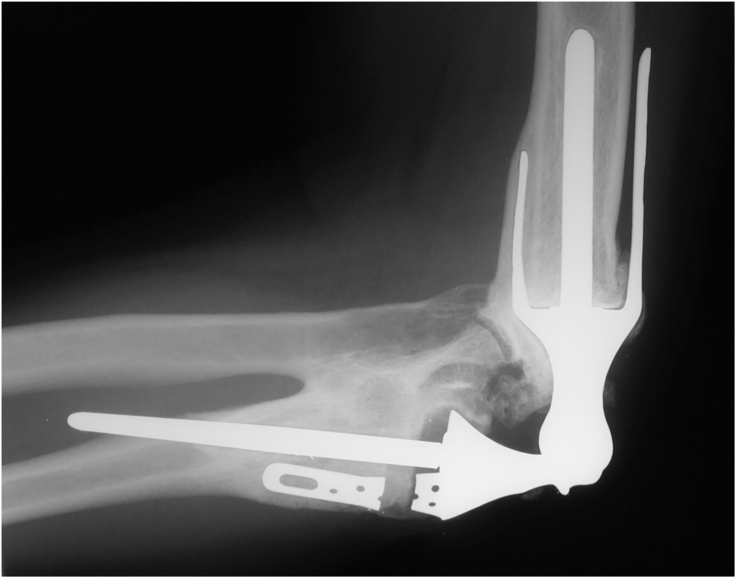
Radiograph of the original custom-made total elbow arthroplasty implanted in 1970. The humeral component exhibits both anterior and posterior flanges, and the ulnar component shows a fractured flange. The ulnar stem is positioned eccentrically, with the shaft component tracking extraosseously along the cortex. Only this archival lateral projection was available; an anterior-posterior view from the initial treatment period could not be retrieved.

A right-hand–dominant man suffered a high-energy left elbow fracture in 1969. At the age of 18 years, he underwent implantation of a custom-made linked TEA in 1970 (Fig. 1). The following 2 subsequent revisions were performed: the first in 1985 and the second in the early 1990s, both using a Coonrad–Morrey prosthesis.

**Figure 2:**
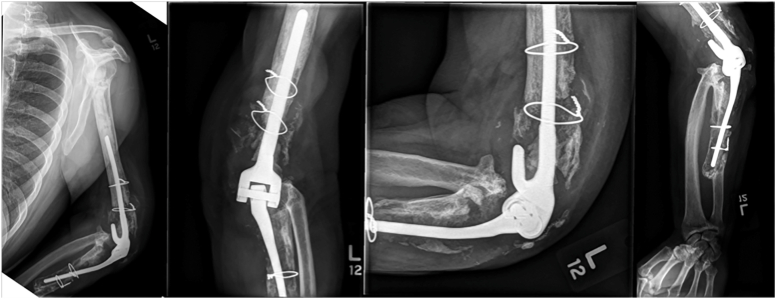
Pre-operative radiographs (2017) showing a ballooned proximal ulna with severe segmental bone loss, progressive humeral periprosthetic lucency, and a mature radioulnar synostosis.

In 2017, at the age of 65 years, he presented with severe pain and disabling instability. Elbow range of motion was limited to 30° of extension and 60° of flexion, with complete loss of forearm rotation due to a mature radioulnar synostosis. Radiographs demonstrated a ballooned proximal ulna and progressive periprosthetic bone loss (Fig. 2).

The patient had blood drawn for a complete blood count, erythrocyte sedimentation rate, and C-reactive protein. An image-guided aspiration of the elbow for culture was also performed pre-operatively. The blood tests showed no abnormalities and cultures were also negative.

#### 2017 stage 1: humeral allograft–prosthesis composite + temporary radialization of the ulnar component

**Figure 3:**
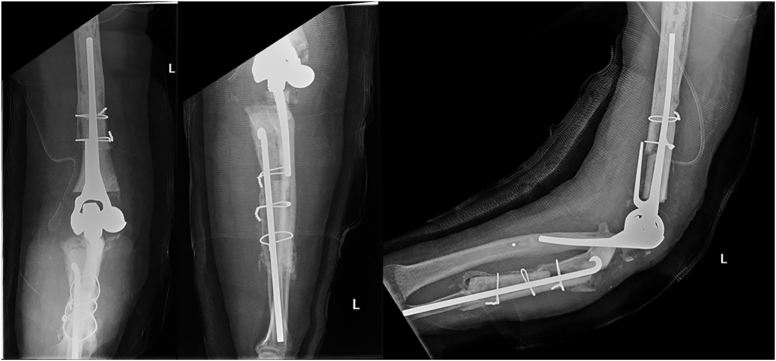
Early post-operative radiographs demonstrating satisfactory alignment. The humeral APC is stabilized with 2 cerclage wires. The biologically reconstructed ulna shows a Rush rod, allograft struts, cancellous graft packing, and 3 cerclage wires. The ulnar component is temporarily implanted into the proximal radius, where the intramedullary cement stopper is visible. *APC*, allograft–prosthesis composites.

Through a posterior approach with a radial and ulnar nerve neurolysis, the loose components were removed. A humeral APC was constructed to restore length and alignment. Since proximal ulnar bone loss made immediate ulnar fixation impossible, the ulnar component of the Latitude EV Elbow Arthroplasty System (Tornier, Bloomington, MN, USA) was temporarily implanted into the proximal radius. This allowed re-establishment of a linked articulation while the ulna was biologically reconstructed. A humeral allograft was used for the distal humeral APC reconstruction. After preparation of the distal humeral APC, cancellous bone was harvested from the humeral head and packed into the deficient proximal ulna around a Rush rod. Cortical struts fashioned from the humeral shaft were then applied to the posterior and radial aspects of the proximal ulna and secured with cerclage wires to provide structural support during biological incorporation. Post-operative imaging (Fig. 3) confirmed satisfactory component alignment.

#### 2018–2020 interval

**Figure 4:**
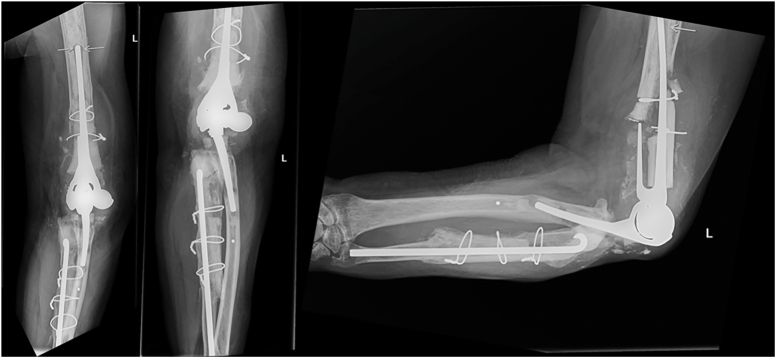
Radiographs from 2020 showing progressive humeral component loosening, with widening radiolucent lines along the proximal stem, loss of integrity of the distal humeral cerclage construct, and increasing apex-anterior angulation of the distal humeral segment. The proximal cerclage wire has eroded into the anterior cortex. The biologically reconstructed ulna remains structurally intact, though new radiolucent lines are visible around the radially implanted ulnar component.

Over the subsequent years, sequential follow-up demonstrated that the humeral allograft healing had failed. By November 2020, imaging showed loss of integrity of the distal humeral cerclage construct, with increasing apex-anterior angulation of the distal humeral segment. The proximal cerclage wire had eroded into the anterior cortex, and widening lucent lines at the proximal stem–bone interface indicated gross humeral component loosening. In contrast, the biologically reconstructed ulna healing had progressed. New radiolucent lines had begun to appear around the radially implanted ulnar component. Infection workup, again including serologic testing and joint aspiration, remained negative, supporting aseptic mechanical failure (Fig. 4).

#### 2021 stage 2: ulnar allograft–prosthesis composite reconstruction and modular distal humeral reconstruction

In 2021, a further revision TEA was performed. A ulnar APC was constructed using an extra-long ulnar component (Discovery Elbow System, Enovis, Wilmington, DE, USA) inserted through a fibular allograft collar and into the biologically reconstructed ulna, which had largely incorporated. The extra-long stem was selected to obtain fixation in good distal ulnar bone stock. During cementation, cement was inserted under pressure into the ulnar canal and into the prepared fibular allograft collar before the ulnar component was passed through the collar graft and into the ulna, ensuring cement support around the proximal part of the ulnar component. Due to the absence of reliable anatomic rotation landmarks and the persistent synostosis, the ulnar component was inserted with the forearm in approximately 40° pronation.

The switch from the Latitude EV Elbow Arthroplasty System to the Discovery-based revision construct was chosen because the Discovery system has a longer off-the-shelf ulnar component than the Latitude EV system, avoiding the need to wait for the manufacture of costly custom components.

The humerus was reconstructed using a modular distal humeral system (Comprehensive SRS, Zimmer Biomet, Warsaw, IN, USA), consisting of a distal body, a 30-mm intercalary segment, and an anterior flange for rotational control. Fixation was augmented with a posterior humeral strut allograft.

#### 2025 follow-up

**Figure 5:**
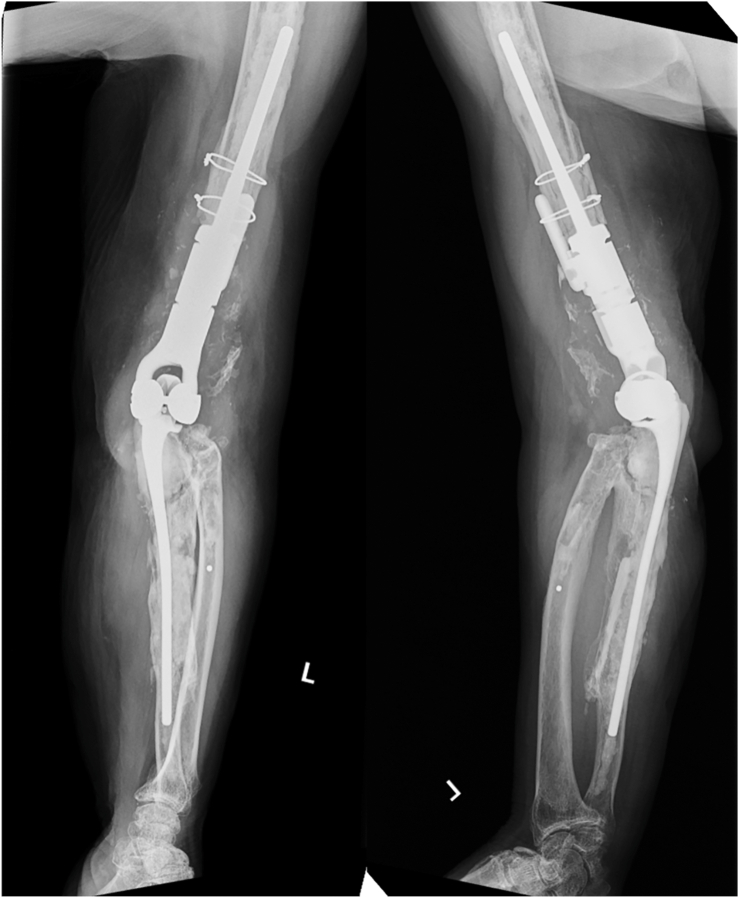
Four-year follow-up radiographs (2025) demonstrating stable positioning of the definitive reconstruction, including the modular distal humeral replacement with strut augmentation of the posterior humeral cortex and the ulnar APC with a long-stem implant seated within a fibular allograft collar. Both components show progressive graft incorporation without evidence of loosening. *APC*, allograft–prosthesis composites.

At 4-year follow-up, the now 73-year-old patient reported mild pain. Elbow extension remained 5° short of full extension, flexion was 90°, and forearm rotation was persistently absent due to mature synostosis. The elbow was clinically stable and the patient was able to perform all evaluated activities of daily living (combing hair, feeding, personal hygiene, putting on a shirt, putting on shoes) without assistance. The MEPS score was 80/100 (pain: 30/45; ROM: 15/20; stability: 10/10; function: 25/25), corresponding to a good result. Radiographs demonstrated stable implants and progressive allograft incorporation (Fig. 5).

## Discussion

Massive bone loss in the multiply revised elbow remains a key obstacle to successful revision TEA. The present case illustrates a staged salvage concept combining temporary radialization of the ulnar component with ulnar reconstruction and definitive humeral reconstruction using APC techniques and a modular endoprosthetic system. The staged strategy was driven by the uncommon constellation of proximal ulnar deficiency, rigid proximal radioulnar synostosis, and a multidecade implant history and was further necessitated by loosening of both the humeral APC and the radialized ulnar component. The first stage focused on biologically reconstituting the ulna to enable definitive reconstruction.

APCs remain a key component of limb-salvage reconstruction in both oncologic and nononcologic elbows with massive bone loss. Morrey et al^14^ demonstrated that, even in a cohort with an average of 5.3 prior procedures, reliable graft-host union and substantial functional improvement can be achieved when adequate cortical contact, biologically active host bone, and stable strut augmentation are provided, yielding radiographic incorporation in 92% of cases. Burnier et al^3^ further demonstrated that proximal ulnar reconstruction can be combined with extensor mechanism reconstruction using a structural proximal ulnar allograft with attached triceps tendon. In their series of 10 revision elbow arthroplasties for combined ulnar bone loss and triceps insufficiency, pain improved in all elbows at a mean follow-up of 45 months, with a mean flexion-extension arc of 95° and a mean MEPS of 76 points; however, 6 elbows required reoperation. In the present case, early ulnar reconstruction with dual cortical struts and cancellous allografting established a biological foundation that later enabled secure placement of a long-stem implant within a fibular allograft collar.

Temporary radial implantation of the ulnar component, required because the ballooned and structurally compromised ulna could not support stable fixation, served as a functional bridge during the first stage of reconstruction. Although driven by the rigid radioulnar synostosis, which effectively reduced rotational forces, radialization may represent a legitimate salvage option even in synostosis-free settings of profound ulnar deficiency.^2^^,^^11^ This maneuver restored elbow function while permitting biological rebuilding of the ulna while maintaining joint alignment. Despite these short-term advantages, the gradual development of radiolucent lines around the radialized ulnar component in this case reflects unfavorable load transfers associated with radial implantation. These findings are consistent with the reports by Lee and by Bellevue et al^2^, both of whom noted that the canal geometry and altered stress distribution of the radius predispose to loosening and polyethylene wear.^11^ Gong et al^6^ reported radial implantation of the ulnar prosthesis as a salvage procedure in 4 revision TEAs with Grade III ulnar bone defects, resulting in painless functional elbows without major complications at short-term follow-up. Collectively, these observations support the principle that radialization is best conceptualized as a temporary stabilizing measure rather than a definitive solution for a failed TEA. Custom components represent an additional option for massive proximal ulnar bone loss in revision TEA. A custom long-stem ulnar component or custom ulnar megaprosthesis may allow the surgeon to bypass deficient proximal ulnar bone and obtain fixation in more distal bone stock. Such an approach could also have been considered in the present case. However, the staged strategy was chosen because the ulna was considered biologically reconstructable, and restoration of ulnar bone stock was desirable for later definitive fixation given the relatively young age of the patient. Furthermore, the cost and manufacturing lead times for custom components can be a challenge in many health care systems. Recent humeral APC data emphasize the persistent challenge of securing reliable graft–host incorporation in complex revision settings. Cheema et al^4^ reported graft–host nonunion in 36% of cases and a 32% revision rate despite the use of compression plating, indicating that humeral composites remain mechanically vulnerable. In our case, loss of fixation after the first stage called for transition to definitive reconstruction with a modular distal humeral replacement, which restored length, alignment, and rotational control and hopefully will prove to be a more dependable foundation for durable fixation in a severely compromised limb.

The final elbow arc of flexion-extension (5°–90°) falls short of the traditional 30°–130° functional range; however, contemporary functional analyses of TEA emphasize that stable motion within a narrower arc may still support independence in most daily activities.^8^^,^^13^ This patient had a long-standing loss of elbow motion from prior trauma complicated by a radioulnar synostosis. His current motion represents an improvement over his previous arthroplasties and he is satisfied with his functional result.

## Conclusion

This case illustrates the following 3 key principles: temporary radial implantation of the ulnar component of a TEA can restore a stable functional elbow and permit biological reconstruction when ulnar bone deficiency is severe; durable fixation of the ulnar component in the proximal radius should not be expected; and staged reconstruction of ulnar bone deficiency and modular distal humeral replacement can reestablish alignment, stability, and functional motion in elbows with extreme bone loss.

## Disclaimers:

Funding: No funding was disclosed by the authors.

Conflicts of interest: Graham J. W. King receives royalties and is a consultant for Stryker Inc. He is an associate editor of Journal of Shoulder and Elbow Surgery Reviews, Reports & Techniques. Any additional authors, their immediate families, and any research foundations with which they are affiliated have not received any financial payments or other benefits from any commercial entity related to the subject of this article.

Patient consent: Obtained.

## References

[1] Aponte-Tinao L.A., Ayerza M.A., Muscolo D.L., Farfalli G.L. (2013). Allograft reconstruction for the treatment of musculoskeletal tumors of the upper extremity. Sarcoma.

[2] Bellevue K.D., Lorenzana D.J., Klifto C.S., Richard M.J., Ruch D.S. (2021). Revision total elbow arthroplasty with the ulnar component implanted into the radius for management of large ulna defects. J Shoulder Elbow Surg.

[3] Burnier M., Nguyen N.T.V., Morrey M.E., O’Driscoll S.W., Sanchez-Sotelo J. (2020). Revision elbow arthroplasty using a proximal ulnar allograft with allograft triceps for combined ulnar bone loss and triceps insufficiency. J Bone Joint Surg Am.

[4] Cheema A.N., Conyer R.T., Triplet J.J., O’Driscoll S.W., Morrey M.E., Sanchez-Sotelo J. (2023). Outcomes of humeral allograft-prosthetic composites with plate fixation in revision total elbow arthroplasty. JB JS Open Access.

[5] Geurts E.J., Viveen J., van Riet R.P., Kodde I.F., Eygendaal D. (2019). Outcomes after revision total elbow arthroplasty: a systematic review. J Shoulder Elbow Surg.

[6] Gong M.-Q., Jiang J.-L., Jiang X.-Y., Zha Y.-J., Li T. (2016). Inserting the ulnar prosthesis into radius as a novel salvage surgery for revision total elbow arthroplasty with massive bone defect. Chin Med J (Engl).

[7] Gonzalez M.R., Okay E., Sodhi A.S., Lozano-Calderon S.A. (2024). Reconstruction of the elbow with distal humerus endoprosthetic replacement after tumor resection: a systematic review of the literature and institutional case series. J Shoulder Elbow Surg.

[8] Haverstock J.P., King G.J.W., Athwal G.S., Johnson J.A., Langohr G.D.G. (2020). Elbow motion patterns during daily activity. J Shoulder Elbow Surg.

[9] Kamineni S., Morrey B.F. (2004). Proximal ulnar reconstruction with strut allograft in revision total elbow arthroplasty. J Bone Joint Surg Am.

[10] Laumonerie P., Granjou J., Tibbo M.E., Massin V., Bonnevialle N., Mansat P. (2023). Midterm outcomes allograft prosthetic composite reconstruction for massive bone loss at the elbow. Orthop Traumatol Surg Res.

[11] Lee D.H. (2016). Radialization of the ulnar component during revision total elbow arthroplasty: a case report. JBJS Case Connect.

[12] Mansat P., Adams R.A., Morrey B.F. (2004). Allograft-prosthesis composite for revision of catastrophic failure of total elbow arthroplasty. J Bone Joint Surg Am.

[13] Morrey B.F., Askew L.J., Chao E.Y. (1981). A biomechanical study of normal functional elbow motion. J Bone Joint Surg Am.

[14] Morrey M.E., Sanchez-Sotelo J., Abdel M.P., Morrey B.F. (2013). Allograft-prosthetic composite reconstruction for massive bone loss including catastrophic failure in total elbow arthroplasty. J Bone Joint Surg Am.

